# Physical Exercises in Schools

**Published:** 1913-04-12

**Authors:** 


### PHYSICAL EXERCISES IN SCHOOLS.

### Ceremonial Drill and Violin Playing.

Recently most school doctors have paid some attention to the question of physical exercises. It is realised that such exercises are of great benefit not only in the case of children who suffer from certain nasal and thoracic conditions, but also in the case of children who are anæmic. There is yet a third class of child for whom these exercises are admirably adapted. This is the class to which an experienced teacher has applied the name of "camel." All who have to do with children are familiar with this type, the boy or girl who, while not definitely scoliotic, has habitually a stoop with the head sunk forward, the shoulders hunched up, and the upper part of the dorsal curve exaggerated. This type gains little from the usual exercises given at the schools. The Board of Education has drawn up a syllabus of exercises which are supposed to be undertaken in every school. From a strictly medical point of view these exercises cannot be said to be ideal, but the question, after all, is a much broader one and cannot be wholly decided on orthopædic points alone. It is becoming generally recognised that physical exercises without a definite object in view is, in the case of children at least, a profound mistake. The average child needs to have his imagination stimulated, or the result will be that he carries out his instructions automatically and derives very little benefit from the system, whatever it is, that is prescribed.

Undoubtedly the most beneficial exercise that can be given to the average school child is ceremonial drill, organised with some definite object in view. This is now given in many schools with commendable regularity, and the results, as most school doctors know, are excellent. Military drill is even more beneficial, but there yet remains a considerable amount of opposition to its introduction in schools. Our own opinion is that this view is a mistake. The exercise given under a system of military drill has two great advantages which no other system possesses. In the first place, it inculcates habits of orderliness, discipline, and attention in a manner that appeals to the boy or girl. In the second place, it is a graded system that involves no undue hardship on the individual and does not develop one set of muscles at the expense of another. Anyone who wishes to see for himself the immense benefit that even a modified system of military drill can bring to a class need only visit one of the few London schools where it is in use and compare the physique —and let us add the *morale*—of the children who are being educated there with their fellow-scholars in other schools who receive only the benefit of the Board of Education syllabus.

From the school doctor's point of view the matter of physical exercise is largely one of accessory treatment in certain cases. Some education authorities make special provision for special physical drill classes, the members of which are recruited from a group of scholars whom the school doctor thinks ought to have special drill. The majority of these scholars suffer from spinal curvature in a lesser degree than necessitates hospital treatment. On the Continent such classes are regularly given, and are in charge of trained nurses or organisers who have been specially selected because of their proficiency in gymnastics. At Berlin, for example, a number of such classes are given at the Royal Surgical Clinic. The children attending these classes, from two to four in the afternoon, are put through a course of ceremonial drill, with special exercises in certain cases. The time devoted to such exercises and attendances at the clinic is counted as school time—that is to say, the scholar counts these attendances as school attendances in the ordinary sense of the term. With us the advance is not yet so far. Classes are given only at the schools and are usually under the supervision of class teachers who have had no special training in gymnastics and who are therefore unable to correct faulty positions, however capable they may be to see that the ordinary prescribed evolutions of the drill-book are carried out. Probably we shall get a change in this direction before very long, when the school nurse, who at present splits her energies in various directions, is told off to supervise various groups of scholars who, while they do not need hospital or direct medical treatment, urgently stand in need of what may be called school treatment. Already, we understand, the London County Council is making a move in this direction, while private schools, notably at Leeds, where the services of an expert Swedish gymnast have been secured, have done pioneering work in this department.

To the exercise class the school doctor will refer cases which are markedly in need of physical culture. Chief among such children are those suffering from respiratory defects, such as defective nasal breathing not due to definite nasal obstruction. The French school doctors have recently called attention to a special syndrome in children who have been operated upon for adenoids—the so-called post-operative nasal apnœa—in which the habit of mouth breathing is perpetuated by the atrophy of certain respiratory muscles. This is a condition which is singularly amenable to cure by properly graded physical exercises, and it is one which is unfortunately very common in schools. Another class of child is the scholar who has slight curvature of the back. In the vast majority of cases this is a curve due to weakness of the musculature of the spine; in a smaller number of cases the curve is definitely due to faulty attitudes assumed in certain school occupations, the chief of which undoubtedly is violin playing. Much of the harm done by bad desks and faulty attitudes in writing, about which so much has been said, is counteracted by the regular swimming lessons which are now part of the ordinary routine in certain schools, but the scoliosis resulting from violin playing is usually of a much more severe form than that engendered by any other school occupation.

